# Hospital and Institutional News

**Published:** 1916-09-16

**Authors:** 


### HOSPITAL AND INSTITUTIONAL NEWS.

### THE QUEEN AND THE LONDON SCHOOL OF MEDICINE FOR WOMEN.

It is announced that the Queen will open on October 2 the extension of the London (Royal Free Hospital) School of Medicine for Women, in Hunter Street, Brunswick Square. This extension was in contemplation before the war, but was postponed; it has now been rendered imperative by the very large increase of women students in London. In December, 1914, the Council published an appeal for funds, and in sixteen months the whole £30,000 asked for was subscribed and building operations were begun, which are now almost completed. The flourishing condition of the Royal Free Hospital School is paralleled by the strides that are being made at Edinburgh, where the University authorities, as was foreshadowed in these columns some time ago, have thrown open the University classes to women students. A considerable increase is expected over the one hundred and fifty women students who were attending extramural classes before this removal of University restrictions.

### BRITISH RED CROSS STAMP.

The King, whose interest in and patronage of philately are well known, has accepted a special die proof for his collection of a special stamp issued by the Philatelic War Fund, 151 Strand, W.C. The design, which His Majesty has personally commended, shows Britain taking up the sword of Justice. The engraving and printing have been entrusted to Messrs. Perkins, Bacon and Co., printers of the first postage-stamp ever issued, the famous "Black Penny" English stamp of 1840. The stamps are printed in six different colours, but the price of all the varieties is the same—namely, one penny. The proceeds are to be allotted to the British Red Cross Society and the Order of St. John of Jerusalem in England. It is stated that all the Allies except Serbia and Montenegro have now issued special stamps in aid of war charities.

### THE WAR AND THE MEDICAL DEFENCE UNION.

The annual meeting of the Medical Defence Union will be held at the Royal Society of Medicine on September 21, and the annual report has been circulated to the members of the Union. The usual record of hard and successful work, thoroughly well done, is disclosed by the report, which has been somewhat abbreviated owing to war conditions. The table of cases undertaken shows that libel and slander actions (on professional questions) are diminishing in the most gratifying manner, as also are cases of unqualified practice and impersonation and malapraxis actions. On the other hand, partnership disputes, arbitrations, and matters arising out of the National Insurance Acts tend steadily to increase, as do legal expenses and other disbursements of the Union. While congratulating the Union on another year of good work for its members, we could wish that greater frankness had been shown in the report on the matter of finance. Only by careful dissection of the accounts is it possible to ascertain that the Union was actually out of pocket on the year's work: this should have been pointed out to the members, in our opinion, as it is obviously a fact which closely concerns them. One reason for this excess of expenditure over income is the Union's activity in undertaking prosecutions which are not for the benefit of individual members, but for that of the whole medical profession. This entitles the Union to the gratitude of all doctors, and to their practical sympathy by joining; but it is not "business" if it means a balance on the wrong side in the accounts.

### AN ASSISTANT MEDICAL OFFICER'S PEERAGE.

It is officially announced that a writ, dated September 8, 1916, directed to Gervase Disney Alexander (lately assistant medical officer at Bodmin Mental Hospital), summoning him to the Upper House of Parliament by the name, style, and title of Gervase Disney Alexander de Cobham, Chevalier, has been passed under the Great Seal, pursuant to warrant under His Majesty's Royal Sign Manual. Five months ago The Hospital recorded the intention of determining the abeyance of the barony, the original claimant being Dr. Reginald Gervase Alexander, father of the present peer, formerly senior physician of Halifax Royal Infirmary, who died on February 19 last. Now that the new peer has been summoned to the House of Lords we can only reiterate the hope, previously expressed, that he will realise his responsibilities to the medical profession, and in his new and more exalted sphere of influence be ready to act as spokesman not merely on matters affecting his former professional colleagues, but in particular as regards the position and prospects of the assistant medical officers in asylums, whose needs and conditions of work were once his own. If the Association of Assistant Medical Officers in Mental Hospitals has survived the war, we hope that it will approach Lord Cobham and invite his co-operation in its recent endeavour to improve the status and prospects of the assistant medical staff in our mental hospitals.

### TWO ROGUES SENTENCED.

The public have during the past week been protected against two dangerous irregular practitioners for periods of six and twelve months respectively. The lighter sentence of imprisonment has been bestowed upon one Thomas Coffey, who has not only practised in another man's name for six years at Swansea, where he obtained a Poor-Law appointment and a public vaccinatorship, but even had the effrontery to take a commission in the Royal Army Medical Corps. It was only when the real Simon Pure also applied for a commission that the fraud was detected. Inasmuch as this man has forged death certificates said to be over a hundred in number, besides obtaining money under false pretences, his sentence appears exceedingly light.

The other detected quack was a music-teacher with no medical training or experience of any kind, who treated everyone on the uniform system of three days' complete starvation. For four months he does not seem to have killed anyone by this delightfully simplified science of medicine; but it was naturally only a matter of time before a victim failed to survive the régime. It was shown that the unfortunate lady in question suffered from heart disease, a fact that the accused did not attempt nor was able to ascertain. In this case the judge made some stringent censures of the quack, and intimated that only the jury's recommendation to mercy and the lack of an element of robbery deterred him from ordering a severer sentence than twelve months' hard labour.

### ALLEGED CHARGES TO WOUNDED IN HOSPITAL.

A signed letter has appeared in the *Glasgow Citizen*, in which the writer says: "In the course of one of my recent visits to wounded friends in hospital, I was rather surprised to learn from them that on being discharged they are debited with certain charges by the hospital authorities for treatment received (be it under the guise of 'extras' or otherwise), which act is naturally like a lump sticking in their throats." What is the basis of this statement? To what does it refer? and how can what appears to be an unfortunate misconception have arisen? The fact that the writer, Mr. P. S. Henderson, found the impression on his visit makes it desirable for the authorities of the Glasgow hospitals to explain what the circumstances really are.

### SIR JOHN FURLEY'S NEW SUGGESTION.

There must be few, if any, living men to whom the Red Cross movement in this country owes so much as to that fine old veteran, Sir John Furley, and any suggestion made by one of so ripe an experience is bound to receive sympathetic consideration. In a letter to the *Times*, Sir John puts forward a scheme for the establishment of a "Hospital and Ambulance Museum," or rather, revives an old scheme of his for such an establishment. He tells us that many years ago he and the late Lord Wantage nearly brought such a plan to fruition. The object held most in view is to obtain standardisation by international agreement of ambulance stretchers, waggons, and other materials. Such an arrangement would obviate much suffering to the wounded whenever they have to be transferred from the vehicles of one nation to those of another. As matters stand, a wounded man cannot be thus transferred on his stretcher because the waggons of one nation will not take the stretchers of another. Another purpose of such an exhibition is to compare the values of suggested improvements, to further expert criticism, and to dissipate the ignorance on ambulance *matériel* which Sir John finds often existing in official minds. As might be expected, he has found a sympathiser in Mr. H. S. Wellcome, whose medical museum in Wigmore Street is such a triumph of organisation. If Sir John proposes to add a historical section to his project, it may be suggested that much of the ambulance equipment of the British Army at the outbreak of war might be placed in it as a warning and an example.

### MEDICAL ARRANGEMENTS IN MESOPOTAMIA.

Discontent with the medical as with other arrangements and the direction of the Mesopotamian Expedition is growing and becoming steadily more vocal. How much substance there is in the complaints against the Indian Government's management may perhaps come to light when the Commission now sitting issues its Report; but it certainly seems on the information available from letters to the Press that the medical arrangements have been greatly inferior to those on the Western and Mediterranean fronts. Writers in the *Daily Mail* have denounced the green goggles supplied to the troops in Mesopotamia as useless and even actually harmful: the War Office declines to disclose the name of the officer who passed the glasses for issue. The *Morning Post* is equally scathing on the treatment of the sick and wounded at Coonoor and on the breakdown of medical and hospital services in Mesopotamia. The name of Sir William Meyer (Financial Adviser to the Viceroy) is freely bandied as the person primarily responsible for the shortcomings of the Medical Services. The *Morning Post* wisely declines to condemn this official on the strength of mere bazaar gossip, but would like to see responsibility fixed and an example made of the culprits: even hanging has been mentioned as the suitable penalty.

### THE FREEMASONS' WAR HOSPITAL.

The Freemasons' War Hospital is now ready for receiving patients, and a representative of The Hospital who visited the institution a few days ago found the wards all prepared and the beds all "made." The premises which the Masonic Nursing Home Committee—acting on behalf of the Masonic craft, which has so liberally subscribed towards the equipment of this institution—has secured are familiar to readers of The Hospital as the Chelsea Hospital for Women in Fulham Road. This most suitable building has, of course, been entirely redecorated and refurnished for its new purpose, and it has been found possible to increase the number of beds to sixty without the slightest suspicion of crowding. In former days the Chelsea Hospital for Women had accommodation for forty patients. In the small wards an electric reading-lamp is fixed behind each bed, and in the large wards there is a reading-lamp between each bed. The operating theatre on the top floor is well lighted and fully equipped; indeed, the whole institution is admirably adapted for use as a war hospital. The institution is under the patronage of Lord Ampthill (Pro-Grand Master of English Freemasons), Mr. T. F. Halsey (Deputy Grand Master), the Hon. Arthur Stanley, M.P. (Provincial Grand Master, Lancashire, Western Division, and Chairman of the Executive Committee of the British Red Cross Society). The idea that the beds will be reserved for sailors and soldiers who are Freemasons is, of course, erroneous.

### ARE AUSTRALIANS UNHAPPY IN BIRMINGHAM HOSPITALS?

A [illegible]ECENT article in the *British Australasian* has caused a flutter in the Birmingham dovecots by declaring that "the great majority of complaints from Australian soldiers who, for one reason or another, are unhappy in British hospitals, come from Birmingham. Seldom, indeed, have we heard a good word for a hospital there, and almost never have we heard a bad word for a hospital in Manchester." It hardly needed this comparison to point the charge, which, on quotation in the Bir-[illegible]ham papers, brought forth an anonymous [illegible] in support of the allegation, and declared [illegible] the discontent was not with all but with one [illegible]ital in particular. This is tantalising, for if [illegible]harge concerns one hospital in particular, [illegible] is a good opportunity for investigation. It is [illegible] the accusers to name it, so that no hospital [illegible]y hide a guilty head amid the chorus of gratitude which has arisen in the form of further letters [illegible] "contented Australians" in the 1st Southern [illegible]eral, 1st Birmingham War Hospitals at Rubery and Rednal, the 2nd B. W. Hospital, Northfield, the General Hospital and the Queen's. A letter of gratitude has also appeared on behalf of Australians in the Birmingham War Hospital, Hollymoor. It is signed by Sergeant J. H. Daly, who says that he has been asked to write. Unless a definite charge is made against a particular hospital, the article and the correspondence which has followed it only tell us that there have been grumbles. As to whether they are justified or not the public is still in the dark. The *British Australasian* must either give institution, chapter, and verse, or take the responsibility for creating an atmosphere of suspicion which anonymous letters of gratitude are not sufficient to allay.

### CAMBRIDGE AND DISTRICT WORKERS' HOSPITAL FUND.

THE Cambridge and District Workers' Hospital Fund lately held its fifth annual meeting at Addenbrooke's Hospital. The committee are conscious that similar organisations in other districts, particularly those where munition work is in progress, show increased results, whereas in the case of the Cambridge Workers' Hospital Fund there is a considerable decrease as compared with previous years. It is accounted for by the large number of male contributors called to military service, whose places as contributors it is difficult to fill. The Convalescent Fund has again accomplished good work in sending seven members to convalescent homes, in supplying thirty-three with surgical appliances, and defraying cost of special treatment for four special cases at their own homes. The total receipts for the year were £481 14s. Of this sum, less £13 for expenses, Addenbrooke's Hospital received, including a special grant, £375. The remainder, save £30 transferred to Convalescent Fund, was divided between two local Nursing Associations and the Royal Surgical Aid Society. At the Wakes Fund Committee meeting in August, Colonel Harding suggested that, as so many of the male contributors had been called to military service, perhaps some of the ladies who had taken their places in their occupations would also step into the breach and subscribe to the Workers' Fund. Only in some such way can the hope of raising £1,000 a year for Addenbrooke's from the Workers' Fund be realised.

### THE DOCTOR'S BILLS OF PUBLIC OFFICIALS.

A SOMEWHAT ridiculous situation has arisen in Blackpool, where the Town Council's decision to pay the doctor's bills for the chief constable and his family, on the ground that the sickness in his household was due to the insanitary condition of his residence, has led to the application by two constables for the payment of their sickness expenses. This has been refused, on the assurance of the medical officer that, unlike the chief constable, the other two applicants had not contracted disease at the police buildings, where the chief constable lived, and the men did not. The chairman of the Watch Committee has pledged his word that ordinary constables will receive the same consideration as their chief, but as the latter and his family seem to have been the only persons affected by the bad condition of the drains, it is difficult to see, these once put right, how anyone else can claim relief from the Town Council.

### WOUNDED SOLDIERS' POCKET-MONEY.

IT has never been explained why there should be so much difficulty in providing the wounded soldiers in the voluntary hospitals with a small instalment of their pay for such things as stamps, shaving, tobacco, and other minor necessaries. In the early days no cash at all was advanced; later a preposterous system was invented under which a few pence could be obtained for specified purposes from the medical officer in charge, upon the signed requisition of the patient, but as the medical officer had to recoup himself by application to a score of regimental headquarters all over the country this system could never be worked with a good heart and was soon dropped. Then came an arrangement under which every month the administrator of the voluntary hospital advanced a few shillings to patients desiring the accommodation, and was repaid by his parent military hospital. This worked excellently and everybody was apparently satisfied; but now, without any explanation, things have reverted to the bad old days of entire dependence upon charity, and the men are allowed no money at all. Early in the war people were tumbling over each other to provide small comforts for the wounded, and such things as postage stamps, stationery, and cigarettes were supplied in profusion; but now, either because the novelty has worn off or because calls upon private resources have become too numerous, our soldiers have often to go short.

### RAPID ACTION UNDER WAR CHARITIES ACT.

THE Charity Commissioners have taken over the control of five war charities, and their powers are to be exercised in the following cases: The French Relief Fund, Our Own Boys' Day Fund, the Counties Rest Homes for British Soldiers, the Belgian

Soldiers' Fund, and Le Berceau Fund. Two of these charities are referred to in the report of the War Charities Committee as having been conducted in an unbusinesslike or otherwise unsatisfactory way. The French Relief Fund was never in a position to claim the approval of the French Embassy in London, and was severely criticised by *Truth* in December 1915, when the cost of administration was said to be as high as 13 per cent. However, many prominent people allowed their names to be used, and about £130,000 was collected, of which it is understood a balance of £42,000 has not yet been expended. The Belgian Soldiers Fund is the next in order of magnitude, and appears to have been a one-man or rather a one-woman concern, having been worked by a Miss S. Carey, without the assistance or control of any committee, who collected £32,000 and a large quantity of gifts in kind in the first eight months of the career of the charity. Miss Carey's good faith is not in question, but the Charity Commissioners evidently think the organisation can be more efficiently managed. The other three charities are apparently small concerns, *mala-fides* are not alleged in the case of any of the five names on the list, and it must be remembered that overlapping as well as faulty administration is, in the eyes of the new legislation, an undesirable feature. The text of the Charity Commission's Memorandum on the Act will be found on another page of this issue.

### FATAL EXPLOSION AT AN INFIRMARY.

A disastrous explosion, resulting in the death of one officer and serious injuries to another, occurred a few days ago at Bermondsey Infirmary, which is situated at Lower Road, Rotherhithe. It originated in the refrigerating chamber of the general stores, where the ammonia plant exploded. Fortunately, the building is situated some short distance away from the infirmary, which escaped, but the store windows and doors were blown out, and the building, with its contents, were seriously damaged. A plucky attempt was made by Dr. Cheal, the assistant medical officer, and by the steward to rescue the injured men, but they were overcome by the fumes. Happily, there was no fire after the explosion. These unfortunate accidents are rare in hospitals and infirmaries.

### THE STAFFING OF MILITARY HOSPITALS.

Considerable difficulties have been experienced in retaining in military hospitals sufficient staff to cope with the ordinary duties of the institutions. The Southwark Board of Guardians have such an experience with regard to their infirmary, which has been taken over by the War Council as a military hospital. The board a month ago decided that they would not appeal to the local tribunal for the exemption from military duty of their stoker. In the month they made every effort to obtain the services of a suitable substitute, and absolutely failed. They were then faced with the situation that if they did not get the exemption of this officer they would be without one altogether, with a prospect of having to ask the two others to work seven days a week, twelve hours a day, a prospect that was not likely to appeal to them, nor was likely to be agreed to. Failing the men agreeing to these conditions, the board had to meet the situation as displayed to them by Major Bruce, the medical superintendent at the infirmary, of being without the necessary hot water with which to bath their wounded soldiers, and not being able to run their laundry. The board, when faced with these facts, had to give way, and instruct their clerk to apply at the Southwark Tribunal for the exemption of their stoker, and also for two porters engaged in checking goods received at the hospital. There is a great dearth of men suitable for filling these positions, and consequently it is essential that those remaining should be retained in order that the work of the hospitals shall not be retarded.

### THE RECOVERY OF THE SCHOOLS.

The recent arrangements in Liverpool for the reception of wounded included the conversion of seven elementary and two secondary schools to hospital purposes. While it is satisfactory to learn that one of the secondary schools has not been required, and that there have been only a few wounded in the other, it is interesting to learn that the military authorities are vacating the buildings a week before the re-opening takes place. It is to be hoped that this plan is being widely adopted, for our satisfaction in knowing that the numbers of wounded during the progress of the offensive in France are smaller than were expected, and prepared for, can only be increased when we learn that one of the results in this country is the re-opening of schools which had been commandeered. To provide for the wounded by a process which involved the disorganisation of education was never a counsel of perfection, and the sooner that it proves unnecessary the better, not only for the children, but also for the men themselves.

### THE FOUNDER OF THE OXFORD EYE HOSPITAL

The death of Mr. R. W. Doyne, F.R.C.S., at the age of fifty-nine, removes the founder of the Oxford Eye Hospital, and one who was constantly prominent in the endeavour to promote post-graduate teaching in ophthalmology in this country. Himself an Oxford graduate, who took his clinical courses at St. George's Hospital, Mr. Doyne began his professional career in the Naval Medical Service. After abandoning this he devoted himself to the study of eye diseases, and was for twelve years Reader in Ophthalmology in the University of Oxford. He got up Long Vacation courses for qualified practitioners, and was active in promoting the Diploma in Ophthalmology, a distinction without which, it was hoped, no surgeon would in time be regarded as eligible for an appointment on the (ophthalmic) staff of any leading hospital. So far this aspiration has not been realised; but the principle of higher degrees or diplomas in special subjects as necessary qualifications for teaching or hospital appointments in those specialities is one that has by no means been indefinitely shelved, and may easily come within the sphere of practical medical politics in the near future. Mr. Doyne was also senior surgeon to the Royal Eye Hospital, London; but it is his work at Oxford by which his memory will be mainly kept green.

### MEDICAL TREATMENT OF SOLDIERS' DEPENDANTS.

The arrangements for supplying free medical advice and medicines to the necessitous dependants of sailors and soldiers came to an end on the last day of August. The scheme came into operation shortly after the outbreak of war, and all over the country doctors undertook to give medical advice to dependants of men serving with the Colours, and pharmacists undertook to dispense the prescriptions free of charge, the actual cost of the drugs being defrayed by the National Relief Fund Committee. The primary reason for the discontinuance of the scheme is that the duties of the Fund relating to naval and military distress have now been transferred to the War Pensions Statutory Committee, which does not see its way to provide the funds necessary for the continuance of the scheme. Apart from this fact, however, there is hardly the same need for relief of this kind as there was in the earlier part of the war, since for the most part there has been such an improvement in the circumstances of the dependants of sailors and soldiers that few of them can be described as necessitous. But cases will no doubt arise which will call for special treatment, and it is to be hoped that these will not be neglected by local relief committees. Some notion of the extent to which dependants benefited by the scheme can be obtained from the fact that during the first nineteen months of its operation over 330,000 medical books were issued. The Duke of Devonshire, as chairman of the Naval and Military Dependants (Medical Treatment) Committee, has sent a letter of thanks to the medical men and pharmacists who gave their services in this connection.

### THE FUTURE OF WINSLEY SANATORIUM.

At last there seems some likelihood of the future of Winsley Sanatorium being determined on the basis of its transference to the Charity Commissioners. As a result of prolonged negotiations the Commissioners have now drafted a plan for the consideration of the governors of the sanatorium, the Bristol and Bath City Councils, and the Wilts County Council. The result of a special meeting of the board of management of the sanatorium was the passing of a resolution approving the Charity Commissioners' scheme, and asking them to establish a scheme of administration on the lines proposed in their draft. Briefly, the scheme vests the property of the sanatorium, with certain exceptions, in the "Official Trustee of Charity Lands," to be managed by twenty-four governors, who will be the trustees; thirteen will be appointed by Bristol, eight by Wilts, and three by Bath. The representatives will include members of the Insurance Committees. The executive will consist of twelve—six from Bristol, four from Wilts, and two from Bath, with Alderman George Pearson as first chairman. In spite of criticism, the scheme, as stated, was approved. The Bristol and Bath City Councils and the Wilts County Council have now to express their opinions upon it. It is to be hoped that the precedent of sinking differences begun by the board of the sanatorium will be followed by the local authorities.

### AN OLD GRIEVANCE RESTATED.

There was a discussion at the last meeting of the Chesterfield Board of Guardians on the abuse of hospitals and workhouse infirmaries by persons who come under the National Insurance Act. The Chairman stated that a man recently came into their infirmary and remained there for fourteen weeks. He promised to repay the Guardians a portion of the cost of his maintenance when he left; but although he drew £7 in sick pay, he spent it all and did not give a copper to the funds. By such means as these insured persons were securing sick pay and institutional treatment free of cost at the public expense, which, said the Chairman, was a disgrace that should be altered at the earliest possible moment. A resolution was passed asking for an amendment of the National Insurance Act to protect hospitals and institutions against those abuses.

### THE GERMAN HOSPITAL REPORT.

In looking through the pages of the report of the German Hospital, Dalston, one experiences a curious feeling in reading that the institution continues to claim the protection of His Majesty the German Emperor and His Apostolic Majesty the Emperor of Austria and the patronage of a dozen Teutonic principalities and powers, including "Their Magnificences", the Senates of Hamburg, Lübeck, and Bremen. This academic guardianship is, of course, harmless enough, but one wonders if an English hospital, situated in Berlin, would be allowed by German popular sentiment to announce at the present time that its work is under the protection of the British Royal Family and the patronage of Their Beatitudes the Corporation of Grimsby and the Bermondsey Borough Council? But the German Hospital, established primarily for German-speaking nations, has always so freely extended the benefits of its excellent organisation to English and other nationalities in cases of accident and emergency that one hesitates to add to the difficulties of the committee by anything approaching adverse criticism except for a more concrete reason than this. It is observed that the total expenditure exceeded the income in 1915 by £3,792, a serious setback to the result in 1914, when there was a surplus of £1,936.

### THE MEDICAL SUPERINTENDENTSHIP OF CLAYBURY.

The retirement, already announced, of Dr. Robert Armstrong Jones from the post of medical superintendent of Claybury Mental Hospital has been followed by two presentations, one from the staff and another from "16,000 past and present patients." The whole staff, medical and lay, united to present him with a tea and coffee service, when Dr. Ewart, his successor, made a speech, calling attention to the detail on which the improvements had been built up. The patients' gift was a silver inkstand. It will be remembered that the new medical superintendent has worked for eighteen years in association with Dr. Armstrong Jones.

### THE DETECTION OF TUBERCULOSIS CONTACT CASES.

THE examination of "contacts" has been attended with very gratifying results at Birkenhead, according to the annual report of the borough's medical officer. Dr. Marsden says that every effort is made to detect early cases of tuberculosis occurring in those who have lived in close contact with notified cases of consumption, and who have thus run the risk of infection. During last year 158 such persons suffering from suspicious ill-health were medically examined, and among them seven cases of tuberculosis were discovered. In the course of the investigation other unsuspected illnesses and defects were found, and these patients were referred to the proper quarters for treatment.

### THE LOCAL GOVERNMENT BOARD AND A MEDICAL OFFICER'S SUSPENSION.

AT the last meeting of the St. Pancras Board of Guardians, London, it was reported that the law costs to be paid by the Guardians in connection with an inquiry regarding certain charges against a district medical officer amounted to £71 5s. 6d. It appears that charges were made that the medical officer (Dr. Cronin) did not carry out his duties in a proper manner. An inquiry was held by the Local Government Board which resulted in an intimation to the Guardians that they must reinstate the doctor. Some objection was taken by several Guardians to paying the costs, but, as one member pointed out, the Guardians were proved to be in the wrong after a full and impartial inquiry, and therefore they must accept the consequences. It is certainly satisfactory to note that before any Board of Guardians can dismiss their medical officer the matter must have the sanction of the Local Government Board. We understand that now Dr. Cronin has vindicated himself he has handed his resignation in to the Guardians.

### A TESTATOR'S THOUGHT FOR NURSES.

THE inclusion of trained hospital nurses in the late Mr. Frederick Andrew's bequest to provide a convalescent home for poor gentlewomen, as announced in THE HOSPITAL of August 12 (Matrons' and Sisters' Department), is interesting, for while those in active work are generally provided for during illness and convalescence by those for whom they work—it was, of course, on this ground that the claim for exemption from the provisions of the Insurance Act was based—middle age often finds them largely unprovided for, notwithstanding the fine work of the Royal National Pension Fund. It is therefore to be hoped that when the convalescent home is founded the intentions of the will may be interpreted so as to extend the benefits of the institution to nurses in need, whether or not they are actively engaged in their profession. In urging this point, we are only reminding the trustees that the conditions of a nurse's life make her chiefly in need of such a convalescent home when her most active days are over. It is also true that after an illness in hospital convalescence in entirely new surroundings, such as Mr. Andrew's Home would provide, is likely to be more beneficial to a nurse than convalescence in the regular home attached to the hospital.

### PATIENTS AND CASES IN HOSPITAL GAZETTES.

THE expectation of a special number of the *1st Eastern General Hospital Gazette*, describing the recent visit of the King to Cambridge, has been disappointed. Instead the O.C. intends to issue a volume of photographs illustrating the occasion. Everyone will regret the reasons which have led to this decision, and the opportunity which the *Gazette* has thus missed of rising to the heights of a special Royal number. The visit, however, is described in unheroic couplets in the latest issue, and a New Zealand patient describes the King's visit to Ward XV. A serial, dealing with an "Episode of the French Secret Service," by Lance-Corporal Hughes D. Collingwood, and an account of a wound from which a patient recovered although a piece of shrapnel was found embedded between the rib and the heart, are the chief contributions, but though the diagram may be interesting, was it necessary to publish the patient's name? It is a good principle not to intrude the personal element into medical or surgical cases. A caricature, a cartoon, some verse, and two short articles complete the number.

### THIS WEEK'S DRUG MARKET.

A QUIET tone pervades the drug market, and so long as the uneasy feeling as regards prices prevails buyers will naturally continue to act very cautiously. The synthetic drugs are especially unsettled, and in view of the fact that we draw a considerable proportion of our supplies from the United States of America, buyers are naturally interested in comparing New York values with those ruling in London. For instance, the price of salicylates in New York is at least 25 per cent. below the price here, and there is therefore excellent grounds for believing that the price in London will be much lower than it is at present. Most of the synthetic drugs are selling in New York at lower prices than they are here. One of the exceptions is phenacetin, which is almost as dear in America as it is in London, where the scarcity has become very acute. In bromides there is no noteworthy change, but makers' prices are well maintained. Hexamine, salol, amidopyrin, aspirin, and formaldehyde all have a downward tendency in value. Barbitone is dearer. Citric and tartaric acids are again rather lower in price. Benzoates are dearer. Acetic acid is cheaper. Emetine is quoted at lower prices in consequence of the reduced value of ipecacuanha.

